# Modeling the Vertical Transport of Copepod Fecal Particles under Nano/Microplastic Exposure

**DOI:** 10.1021/acs.est.5c01967

**Published:** 2025-03-28

**Authors:** Zipei Dong, Wen-Xiong Wang

**Affiliations:** 1 School of Energy and Environment and State Key Laboratory of Marine Pollution, 53025 City University of Hong Kong, Kowloon, Hong Kong, China; 2 Research Centre for the Oceans and Human Health, City University of Hong Kong Shenzhen Research Institute, Shenzhen 518057, China

**Keywords:** microplastics, nanoplastics, copepods, fecal pellets, settling velocity, fluid dynamics modeling

## Abstract

Nano- and microplastics (NMPs) may significantly impact the marine carbon cycle, and fecal pellets produced by the copepods are crucial for vertical carbon transport. In this study, we investigated the effects of NMP size, concentration, and diatom supply on the production and settling of fecal pellets by the marine copepod *Parvocalanus crassirostris*. By employing an aggregation-induced emission fluorescence imaging technique, we visualized the distribution of NMPs in fecal pellets, measured their size and production rate, and developed a fluid dynamic model to simulate the settling process of fecal pellets in the water column. Our results indicated that NPs and MPs exhibited uniform and nonuniform distributions in the produced fecal materials, respectively. NMPs reduced both the size and integrity of copepod fecal pellets. Copepods ingested MPs in the absence of diatoms, but exposure to 5000 μg/L of NMPs decreased the fecal pellet production by 52% in the presence of diatoms due to feeding selectivity. The sinking rates of fecal pellets of varying sizes, as obtained from modeling simulations, ranged from 10.9 to 103.1 m/day. When the proportion of participating polystyrene (PS) reached 50%, the sinking velocity decreased by 34%. Our study provides new insights into the vertical transport of copepod fecal pellets under NMP pollution.

## Graphical abstract

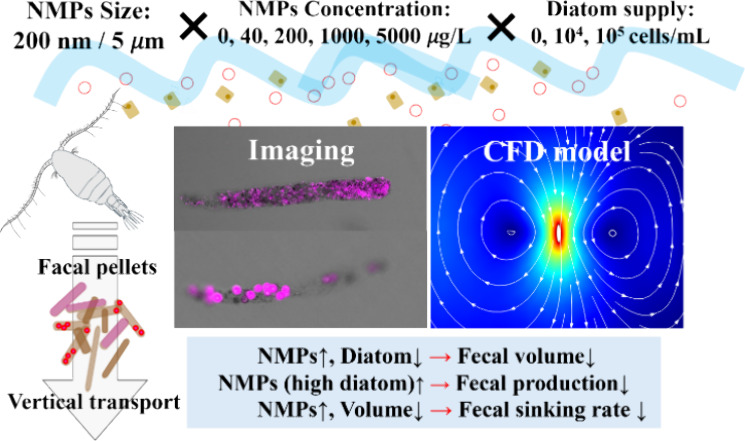

## Introduction

In recent years, nano- and microplastics (NMPs) in the oceans have raised significant concerns regarding their environmental impacts.^1^ According to International Organization for Standardization (ISO 24187:2023), microplastics (MPs) are defined as plastic particles smaller than 5 mm, while nanoplastics (NPs) are those smaller than 1 μm, which may be intentionally manufactured (e.g., microbeads) or result from the degradation of larger plastic materials through mechanical, biological, or chemical processes.^2,3^ Due to the overuse and improper disposal of plastic materials, NMPs are widely distributed and accumulate significantly in the environment.^4,5^ It is estimated that 93–236 kilotons of microplastics are floating on the global sea surface, documented across various marine areas and depths.^6^ Furthermore, both laboratory and field evidence demonstrated that NMPs were ingested by a wide range of marine animals, from zooplankton to whales.^7–9^ The ingestion of NMPs may cause digestive system damage and act as vectors for toxic substances, leading to adverse effects such as feeding disorders, developmental delays, increased mortality, and reduced reproduction.^10–12^ NMPs can potentially transfer along food webs, ultimately affecting humans.^13,14^ These small plastic particles represent a serious threat to the environment and, in particular, to marine ecosystems.

Copepods serve as key energy transfer vectors in marine ecosystems, being among the most abundant primary consumers in the marine food web and dominating the zooplankton community.^15^ Free-living copepods feed on particulate organic matter (POM) in surface waters, including small phytoplankton and organic debris.^16^ Copepods could also pack surface organic biomass into larger, dense, and fast-sinking fecal pellets, which settle to the deeper layers to transport carbon and feed benthic organisms.^17,18^ Zooplankton fecal pellets contribute significantly yet variably to the total vertical particulate organic carbon (POC) flux, with most estimates being less than 40%, although could account for about 94% under productive spring conditions.^19^ Biodriven carbon deposition is estimated to be as high as 30%, compared to the total anthropogenic carbon emissions, and may help mitigate climate change.^20^

However, productive coastal surface waters often overlap with regions polluted by NMPs, leading to frequent encounters and high bioaccumulation with NMPs in marine copepods.^21,22^ Previous studies have shown that copepods can ingest and excrete NMPs.^23–25^ The consumption of NMPs by copepods may facilitate their transfer to higher trophic levels, suggesting one pathway through which NMPs enter the marine food web.^13,22,25^ Copepod fecal pellets also serve as an important nutrient source for fecal-feeding protists, other zooplankton, and benthic organisms.^26^ Incorporation of NMPs into fecal pellets promotes the vertical transport of fecal pellets,^27,28^ and alters their form and surface chemistry, potentially making it easier for other marine animals to ingest NMPs indirectly by consuming these pellets.^29^ Ecological studies show that ballast in fecal pellets, such as calcite and silica contained in algae, affects the density and settling rate of fecal pellets.^30,31^ Similarly, NMPs may affect sinking rates and reduce copepod ingestion rates, selectively rejecting these particles in response to chemical and mechanoreceptors.^32^ Cheng et al.^24^ demonstrated that copepods differ in their ingestion and excretion rates for different sized NPs and MPs, and that this is also influenced by food availability. However, previous studies have struggled to accurately quantify the factors regulating the critical flux of carbon and NMPs, primarily due to the spatial and temporal heterogeneity of NMP distributions and the variability of individual copepods and their fecal pellets.^20,29,33^ Understanding how NMP-mediated changes in copepod fecal pellet production and density influence setting dynamics is critical for improving our understanding of the environmental fate of NMPs and assessing the overall risk they pose to marine ecosystems.

The settling of copepod fecal pellets is influenced by gravity and fluid traction and can be analyzed within the framework of solid settling in a fluid medium.^34^ This process depends on factors such as size, shape, and density, as well as fluid properties like viscosity and density.^35,36^ Larger and denser fecal particles are likely to have higher sinking rates.^30,31^ Most studies have employed settling experiments or modified Stokes equations to evaluate the sinking of fecal pellets.^28,34,35^ While these methods can provide initial predictions of general sinking rate distributions, the lack of accurate quantification and assessment of influencing factors in empirical models limits the potential for extrapolation.^30^ Computational fluid dynamics have been utilized to model the sinking velocities and trajectories of particles under various flow conditions.^37–39^ Numerical models can assess how the properties of fecal pellets themselves influence sinking behavior, which is vital for understanding the factors critical to the vertical transport of carbon and microplastics in marine environments.

In this study, we investigated the production and vertical transport of fecal pellets from a marine copepod *Parvocalanus crassirostris* under exposure to nano- and microplastics. *P. crassirostris* is widely distributed in Hong Kong waters and is one of the most common small copepods, playing an important role in the microzooplankton community of coastal waters.^40^ We visualized and semiquantified the distribution of NMPs in fecal pellets using aggregation-induced emission (AIE) fluorescence techniques and quantified the size and production of fecal pellets under varying NMP sizes, concentrations, and diatom supply conditions. A coupled computational fluid dynamic (CFD) - solid model was then developed to simulate the effects of changes in size and density of copepod fecal pellets under different NMPs mixing patterns (homogeneous vs nonhomogeneous) on setting dynamic and behavior of fecal pellets. Our study provides important insights into the role of NMPs in altering ocean carbon fluxes and their broader ecological impacts.

## Materials and Methods

### Organism Collection and Culture

The collection and culturing procedures for copepods were adapted from established methods.^41^ *P. crassirostris* were collected from Victoria Harbour, Hong Kong, between September and November 2023, using a 300 μm mesh plankton net. To account for the diurnal vertical migration of *P. crassirostris*, sampling was conducted from 20:00 to 22:00 at night, when copepod abundance at surface water was relatively high.^42^ Both the collected plankton and *in situ* seawater were transported in a 5 L clean bottle to the City University of Hong Kong laboratory within 1 h. Upon arrival, large particles and unwanted algae were removed by sieving, and adult female *P. crassirostris* were manually identified and isolated using a stereomicroscope to ensure the selection of only healthy individuals. The copepods were then transferred to aerated artificial seawater (ASW) at 31 ± 1 psu and acclimatized for 24 h under a 12:12 light cycle at 20 ± 1 °C. ASW was prepared using formulated seasalt (Instant Ocean, Blacksburg, VA), which was dissolved in MillQ water and then filtered (0.2 μm), fully aerated to increase the oxygen availability.

The diatom *Thalassiosira pseudonana* was chosen as a food source due to its nutrient-rich profile and size similarity (4–6 μm) to the microplastics (MPs) (5 μm) used in the experiments.^43^ *T. pseudonana* was cultured in f/2 medium under a 12:12 light cycle at 20 ± 1 °C in a light incubator (GZX-150B, Shkytyo, China). Shaker (Orbit 1000, Labnet International Incl., USA) at 80 rpm were added to enhance gas exchange and ensure light uniformity.^44^ During the log phase, *T. pseudonana* was harvested, centrifuged in a benchtop centrifuge (Hitachi CT15RE, Tokyo, Japan) at 1500 rpm for 5 min to remove the culture medium, and redispersed in ASW.

### Fluorescent Nano- and Microplastics

To visualize the distribution of nano- and microplastics (NMPs) in the fecal pellets of *P. crassirostris*, two sizes of aggregation-induced emission (AIE)-labeled fluorescent polystyrene (PS) beads (200 nm and 5 μm) were used to represent NPs and MPs, respectively. These NMPs were specially customized from AIEgens, Ltd. (Guangzhou, China).^45^ The AIE fluorescent probes embedded within the NMPs provide excellent photostability and prevent dye leakage,^46^ allowing for effective tracking of NMP distribution during exposure.^47^ The AIE-labeled NMPs have been tested for toxicity in various aquatic organisms, including plankton,^47^ fish,^48^ and bivalves,^49^ with no direct lethal effects observed. Furthermore, no significant reduction in survival rates was noted in copepods exposed to a maximum concentration of NMPs of 5000 μg/L. The PS material (density 1050 kg/m^3^) exhibits weakly negatively buoyancy in seawater (1030 kg/m^3^), and is one of the commonly used NMPs in pharmaceuticals and personal care products.^50^ In comparison to the typical density of copepod fecal pellets (1140 kg/m^3^), PS is lighter and may interfere with fecal pellet settling.^30^

The difference in size may affect the feeding selectivity of copepods for NMPs. Under laboratory conditions, copepods have been shown to consume NMPs ranging from 50 nm to 30 μm.^41,51^ Previous studies demonstrated the ingestion and accumulation of NMPs by *P. crassirostris* for both sizes.^52^ Although MPs are similar in size and shape to diatoms, they do not fragment *in vivo*, unlike diatoms.^24^ The size of the NPs may fall below the capture limit for *P. crassirostris*, potentially limiting ingestion when NPs are present alone; however, accumulation is observable in the presence of diatoms.^52^

NMPs were stored in suspension at 10 g/L and protected from light by refrigeration (4 ± 1 °C). Concentration data for NMPs were provided by the supplier, and their size distribution, surface charge, and excitation/emission wavelengths of fluorescence were verified in previous studies.^52^ Specifically, the sizes were 218.1 ± 4.6 nm for NPs and 4.63 ± 0.19 μm for MPs, with ζ-potentials of −0.69 ± 0.19 mV for NPs and −4.24 ± 1.01 mV for MPs, respectively. Scanning electron microscope (SEM) images of the surface morphology of the NMPs after sonication are shown in Figure S1. Both NMPs exhibited the same excitation/emission bands at 400/530 nm.

### Experimental Design

To assess the effects of different mixtures of NMPs and diatoms on the size, production rate, and NMP admixture in the fecal pellets of *P. crassirostris*, an experiment with 27 treatment groups was designed, as shown in Table S1. The variables considered included NMP size, concentration, and diatom supply. Two NMP sizes (200 nm and 5 μm) were used, each with a gradient of NMP concentrations (0, 40, 200, 1000, and 5000 μg/L). The treatment group without NMPs served as the control. Concentrations of 40–200 μg/L were considered environmentally relevant, while 5000 μg/L, although exceeding typical environmental levels, may reflect concentrations found in localized hotspots.^21,24,53^ The abundances of small MPs in Hong Kong surface waters ranged from 22,813 to 385160 particles/L at 1–10 μm.^54^ NPs are expected to be even more numerous, as a single MP may be further disaggregated into 10^9^ NPs,^55,56^ reaching orders of magnitude of 10^10^–10^12^ particles/L.^57,58^ High concentration exposure was designed to assess potential effects of NMPs on copepod fecal pellet properties and was acceptable for focusing on effects rather than environmental impacts. Thus, the concentration gradients used in this study were deemed sufficient to cover this range. It is noteworthy that the environmental NMP concentrations are commonly reported in the particle number/volume unit. However, due to the difference of NPs and MPs size, the mass/volume unit is more intuitive and consistent with the conventions of toxicity studies.^59^

Previous studies on the NMP uptake by marine organisms typically focused on only feeding high-concentration NMP, which may overestimate the risk of bioaccumulation. However, the presence of phytoplankton could significantly alter the selectivity of copepods.^23^ Our study remedies this deficiency by exposing copepods to both NMPs and diatoms. Three diatom concentrations were used to simulate varying levels of food availability: no diatoms (0 cells/mL), low concentration (10^4^ cells/mL), and high concentration (10^5^ cells/mL). These concentrations of algae are commonly used for copepod food supply under controlled experimental conditions.^60,61^

A brief diagram of the exposure protocol is shown in Figure S2. The exposure experiment started at midnight (0:00) and lasted for 24 h. Each treatment group had six replicates, with three individuals per replicate. Glass vials were prewashed and rinsed several times with ASW to prevent contamination. Eight milliliters of aerated ASW (31 ± 1 psu) were added to each glass vial, followed by the addition of the NMP suspension, which was sonicated in water-bath sonicator (FUYANG F-020s, China) for 20 min at 20 °C. Diatoms were then added, and the mixture was vortexed and shaken to ensure homogeneous mixing. Copepods were added last to prevent single depletion of NMPs or diatoms before the experiment began. The culture vials were subsequently shaken at low speed (<5 rpm) on a cyclotron oscillator (ZD-85, Changzhou Huanyu, China) under a 12:12 light-dark cycle at 20 ± 1 °C to keep the NMPs and diatoms suspended in the exposure solution. Our light-dark cycle was synchronized with the local environment to ensure that the feeding rhythms of copepods were minimally affected.^42^ Maintaining temporal consistency ensured our results comparable. Copepod survival (as indicated by observable swimming and jumping) was assessed 24 h postexposure, after which formalin was added (1%, final concentration) to terminate the exposure and prevent degradation. The size of fecal pellets was measured within 2 h after the exposure. Although formalin may cause slight shrinkage of fecal pellets, the low concentration and short contact time had little effect on volume (−2.30% in volume, p = 0.63, nonsignificant, detailed in Figure S3), and therefore no correction for shrinkage was made. The glass vials were handled carefully throughout the experiment to avoid damage to the fecal pellets. Finally, the vials were transferred to a dark refrigerator at 4 ± 1 °C for confocal imaging, which was conducted within 24 h.

### Analysis of Fecal Pellets

At the end of exposure, fecal pellets were not removed from the incubation bottles to prevent additional loss and fragmentation. Careful movement and additional resting time allowed all fecal pellets to settle to the bottom. Images of the fecal pellets produced in each treatment were captured using an inverted optical microscope (Nikon Eclipse Ti2, Tokyo, Japan). These images were processed in ImageJ to quantify the length and width of each fecal pellet. For pellets exhibiting curvature, the length was determined as the longest distance along the axis using the folded line approximation, as shown is Figure S3. Each fecal pellet was measured three times, and the average was taken to minimize error. The fecal pellets were observed to be long, thicker in the middle and thinner at the ends, therefore the volume was calculated by generalizing the fecal pellets to an equivalent ellipsoid shape, as shown in Equation 1:

$$V=\frac{4}{3}\times \left(\frac{L}{2}\right)\times {\left(\frac{W}{2}\right)}^{2}$$ 1

Where *V*, L, and W are the volume (μm^3^), length (μm), and width (μm) of the fecal pellets, respectively.

The distribution of NMPs within fecal pellets was visualized using confocal fluorescence microscopy (ZEISS LSM-900 with Objective Plan-Apochromat 20*x*/0.8 M27, Jena, Germany). The excitation/emission wavelengths were set at 405/400–550 nm to capture the fluorescence of the AIE-labeled NMPs, and at 640/600–700 nm to capture chlorophyll fluorescence from diatoms. The images were processed and exported in ZEN (blue edition). Fluorescence intensity was measured in ImageJ (version 1.53q) to semiquantify the NMP content.

### Modeling of Fecal Pellet Settling Using CFD

To simulate the settling process of copepod fecal pellets and evaluate the effects of factors such as size, density and fluid properties, we utilized COMSOL Multiphysics (Version 6.0) to solve the coupled fluid–solid physics problem. The coupled flow-solid modeling captures the trajectory and velocity changes of fecal particles in the water column, providing detailed data that are difficult to obtain under experimental conditions. In the modeling, fecal pellets were represented as rigid ellipsoids (solid domain), while the water column was modeled as a 2000 × 6000 μm rectangle (fluid domain). Initial conditions were set with fecal pellets released 500 μm below the surface of the water column at rest. This design was based on general fecal particle deposition experiments. A built-in CAD design tool was used to create the two-dimensional geometry, with the top and bottom of the water column set as open boundaries and hydrostatic pressure compensation applied to simulate a vertically infinite water environment. No sliding boundaries were applied to the left and right boundaries.

To model the settling of fecal pellets, we accounted for fluid flow in the aqueous phase. Typically, fecal particles experience low velocities when falling in a stationary water column, and the flow field Reynolds number in our simulations is on the order of 10^–3^, indicating laminar flow. The motion of the fluid is described by the Navier–Stokes eqs (eq 2) and the continuity eq (eq 3). The Navier–Stokes equations represent the conservation of momentum in a system in a laminar flow state and include inertial, pressure, viscous, external, and gravitational forces. Conversely, the continuity equation represents the conservation of mass in the system.

$$\rho \left(\frac{\partial u_{fluid}}{\partial t}+u_{fluid}\cdot \nabla u_{fluid}\right)=-\nabla p+\nabla \cdot \left[\mu \left(\nabla u_{fluid}+{\left(\nabla u_{fluid}\right)}^{T}\right)-\frac{2}{3}\mu \left(\nabla \cdot u_{fluid}\right)I\right]+\rho g$$ 2

$$\rho \nabla \cdot u_{fluid}=0$$ 3

where ρ is the density (kg/m^3^), *u*_*fluid*_ the velocity of fluid (m/s), μ the viscosity (N·s/m^2^), and p is the pressure (Pa). I represents the unit matrix.

The fecal pellet in fluid experiences gravitational forces and fluid interactions during settling (Figure 1). The fluid shear and pressure on the surface of the fecal pellet are transferred to the solid fraction, affecting the motion of the fecal particles. The total fluid stress *f* exerted by the fluid on the fluid–solid coupling interface with the fecal pellet is given by eq 4.

$$f=n\cdot \left\{-pI+\left(\mu \left(\nabla u_{fluid}+{\left(\nabla u_{fluid}\right)}^{T}\right)-\frac{2}{3}\mu \left(\nabla \cdot u_{fluid}\right)I\right)\right\}$$ 4

$$F=f-\rho_{solid}V_{solid}g$$

Where *n* represents the outer normal of the boundary. ρ_*solid*_ is the density of fecal pellets (kg/m^3^), *V* is the volume, and g is the acceleration due to gravity.

**Figure 1:**
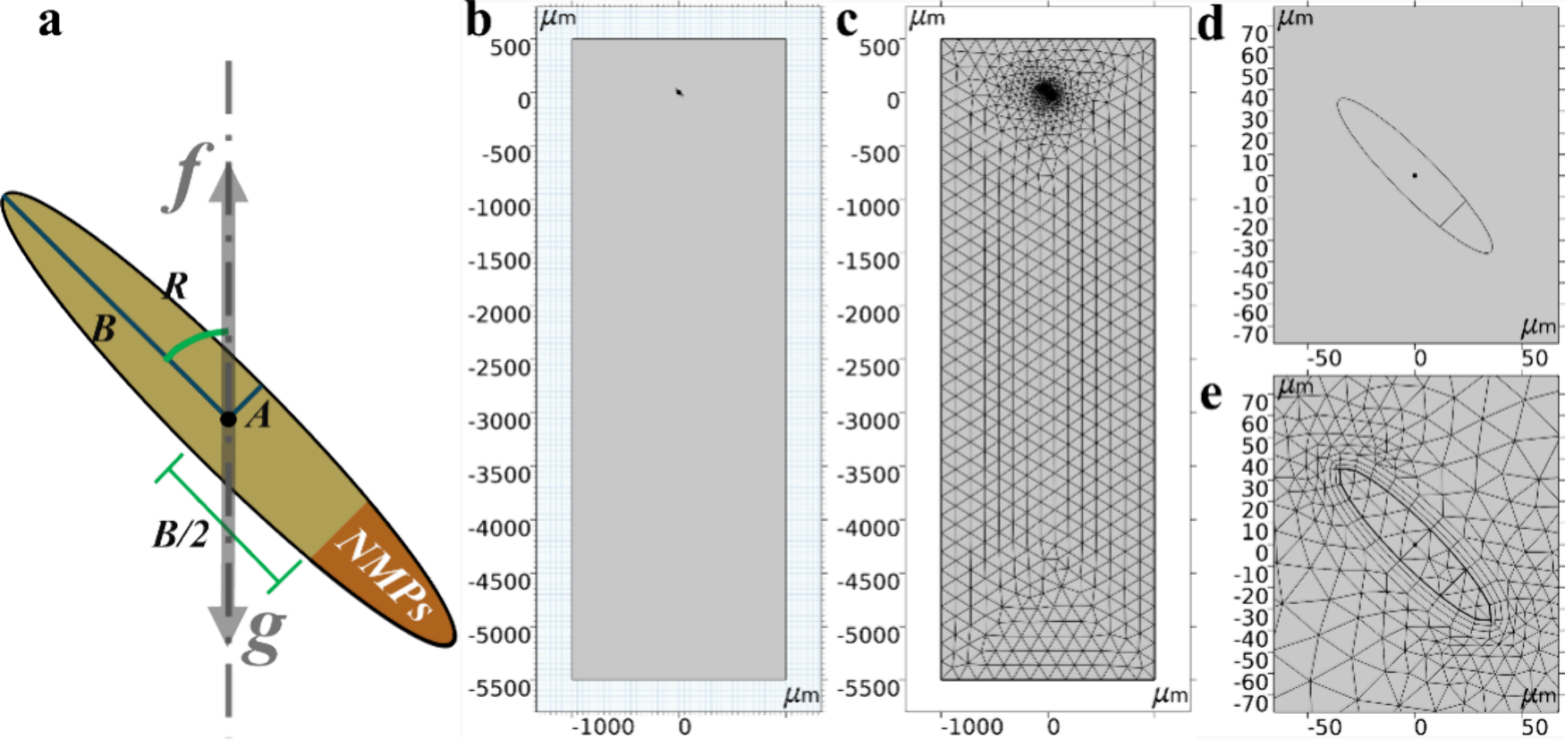
(a) Scheme of a fecal pellet. The size of the elliptical fecal pellet is defined by the short semiaxis A and the long semiaxis B. It is subjected to the trailing force of the water column and gravity. Dark brown color indicates nonuniformly admixed NMPs. (b) Defined geometry including the water column and fecal pellet. (c) Discretized mesh. (d) Geometry localized to the fecal pellet. (e) Mesh localized to the fecal pellet, with the fluid–solid boundary as a quadrilateral mesh and the remainder as a triangular mesh.

At the fluid–solid coupling interface, the fluid velocity is given by eq 5:

$$u_{fluid}{|}_{Interfacing\;boundaries}=(0,u_{solid})$$ 5

Where *u*_*solid*_ represents the velocity of solid (m/s).

We solved the transient model using a detached solution strategy and the finite element algorithm built into COMSOL Multiphysics to compute the displacements and velocities of the fecal particles. To balance model convergence and computational performance, we restricted the maximum time step to 0.001 s and the relative tolerance to 0.001. A triangular mesh was used for spatial discretization, with refinement at the interfaces to ensure computational accuracy. The entire computational domain comprised 1948 cells. Within the fluid domain, mesh deformation was calculated using the smoothing method provided in the moving mesh interface.

$$u_{mesh}{|}_{Interfacing\;boundaries}=(0,u_{solid})$$ 6

Several scenarios were designed to explore the effects of different factors on the fecal pellet settling process. Specifically, these include: 1) Effect of size on fecal pellet settling: The sinking rates of elliptical fecal pellets of varying lengths (50, 100, 200 μm) and widths (10, 20, 30 μm) were simulated. Size settings were based on our observations; 2) Effect of homogeneous incorporation of NMPs in fecal pellets on settling: The sinking process of fecal pellets was simulated for different homogeneous incorporation ratios of PS plastic (6.25%, 12.5%, 25%, 50%). This scenario assumed that homogeneous inclusion would change the overall density of the fecal pellet. For the four parametrization ratios, the densities of fecal pellets were 950, 1050, and 1200 kg/m^3^, with axis lengths and widths set to 20 and 100 μm, respectively; 3) Effect of inhomogeneous admixture of NMPs in fecal pellets on settling: The impact of NMPs on the settling of fecal pellets was simulated under varying densities of NMPs (950, 1050, 1200 kg/m^3^). This scenario was inspired by the distribution of MPs in fecal particles, where NMPs occupied 1/4 of the long axis of the fecal particles, affecting approximately 27.6% of their area. The lengths and widths of the fecal particles were again set to 20 and 100 μm, respectively.

The simulation results included velocity, acceleration, and displacement data of the fecal mass, as well as the velocity and pressure fields of the fluid. We extracted the vertical falling trajectory of the center point of the fecal pellet and analyzed the motion characteristics of the pellet under different conditions. Additionally, one-way sensitivity analyses (2.5% factor change in sinking rate) were conducted for the size and density of fecal pellets and the density and viscosity of water, and the models were validated using a published data set. Details of the sensitivity and model validation process are provided in the Supporting Information (Figure S4, Table S2).

### Statistical Analysis

All statistical analyses were performed using R (version 4.1.3). For between-group comparisons, normality was tested by the Shapiro-Wilk test, followed by the nonparametric Wilcoxon-signed-rank test to determine the significance of between-group differences.

## Results and Discussion

### Distribution of NMPs in Copepod Fecal Pellets

To investigate the admixture of nano- and microplastics (NMPs) in copepod fecal pellets, we visualized the distribution of NMPs in fecal pellets excreted by *P. crassirostris* under varying NMP sizes, concentrations, and food availability conditions (see Figure 2a). The average fluorescence intensity of the fecal pellets was measured to semiquantify the NMP content, as illustrated in Figure 2(b) and Figure 2(c).

**Figure 2:**
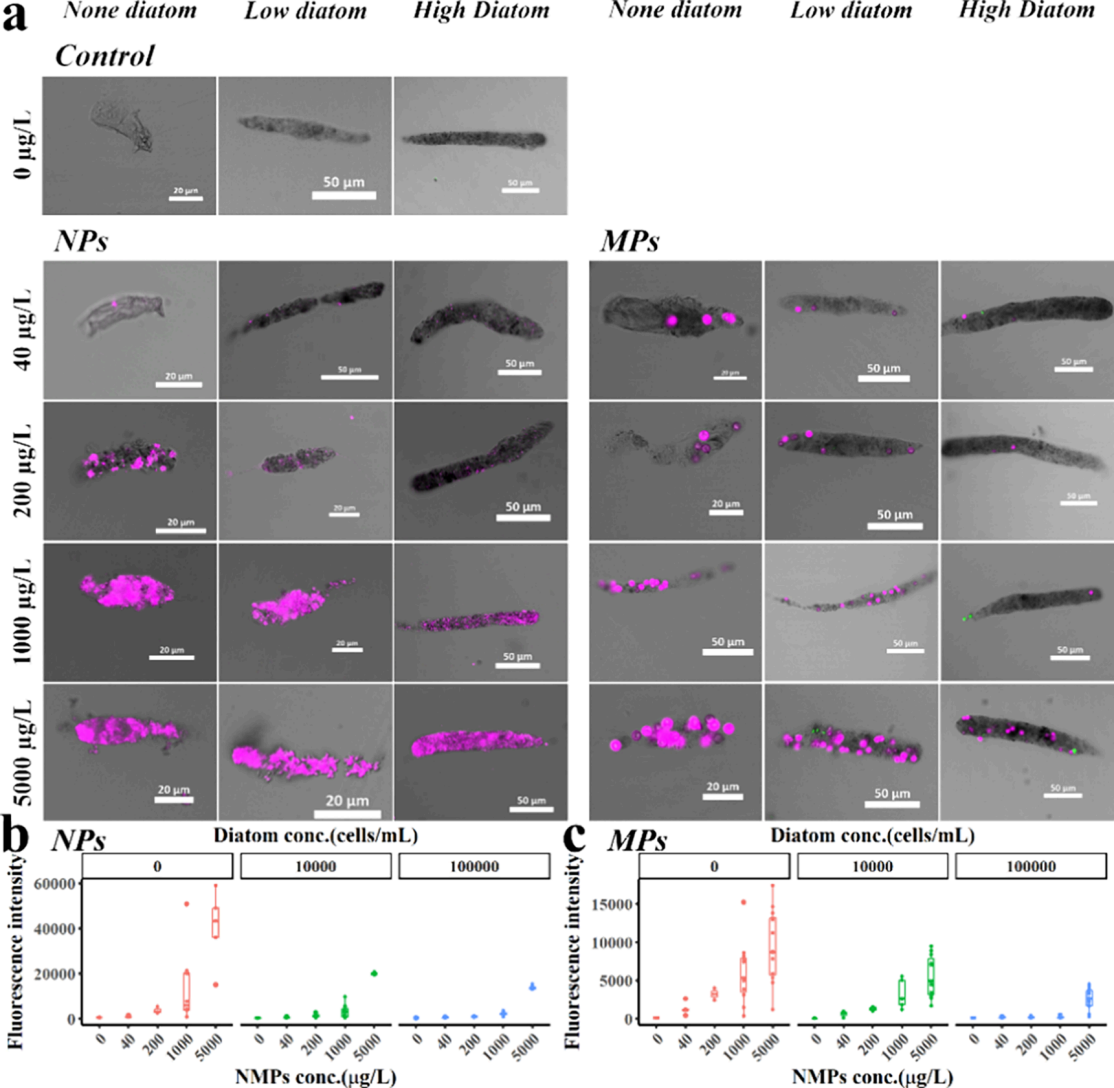
(a) Confocal image of copepod fecal pellets contaminated with NMPs. The presence of fluorescence was observed in fecal pellets produced after exposure to a concentration gradient of the AIE-NMPs/diatom mixture. Pink is fluorescence from NMPs and green is autofluorescence from diatoms; (b) Relative average fluorescence intensity in fecal pellets when copepods were exposed to NPs; (c) Relative average fluorescence intensity in fecal pellets when copepods were exposed to MPs.

To investigate the admixture of nano- and microplastics (NMPs) in the fecal pellets of *P. crassirostris*, we visualized the distribution of NMPs across various sizes, concentrations, and food availability conditions. Fluorescence imaging revealed that both nanoparticle (NP) and microparticle (MP) groups displayed fluorescence from 40 to 5000 μg/L, indicating successful ingestion and excretion of NMPs by *P. crassirostris*. In contrast, no significant fluorescence was detected in the control group. The smaller NPs exhibited a roughly uniform distribution within the fecal pellets, although localized areas of stronger fluorescence suggested partial aggregation during digestion. Conversely, the larger MPs displayed distinct spot fluorescence, and in the 40 μg/L to 1000 μg/L treatment groups, MPs were sometimes concentrated on one side of the fecal pellet. This uneven distribution may result from inadequate mixing in the gut or variable ingestion rates.

Differences in particle size influenced the distribution patterns of NMPs, corroborating findings by Bai et al.,^51^ which noted similar distribution patterns in copepod fecal pellets exposed to NMPs. During digestion, the acidic environment of the copepod gut^62^ may promote the secretion of digestive fluids, facilitating NP aggregation.^63^ Overall, peristalsis and mixing in the copepod midgut likely led to a more uniform distribution of NPs, which could alter overall fecal pellet density. In contrast, the incorporation of MPs resulted in a nonuniform density change.

Copepod fecal pellets serve as vectors for transporting NMPs to other marine organisms. Mo̷ller et al.^64^ reported that *Oncaea* spp. and harpacticoid copepods feed on fecal pellets, while Cole et al.^29^ demonstrated that *Calanus helgolandicus* could ingest NMPs from the fecal pellets of *Centropages typicus*. Additionally, mesopelagic fish are known to primarily consume fecal pellets.^65^ However, the small size and uniform distribution of NPs heighten the risk of reabsorption, particularly by protozooplankton with narrower prey size spectra.^66,67^

Stronger fluorescence in fecal pellets correlated with increasing NMP concentrations, indicating higher NMP levels. This increase suggests potential losses in digestive efficiency, as Cole et al.^12^ demonstrated that NMP exposure can negatively affect energy supply in copepods, leading to reduced lipid accumulation and developmental delays. A decrease in carbon biomass intake may also lower reproductive output.^68^ However, fluorescence measurement may be hindered by digestion residues in fecal pellets, complicating accurate quantification of NMP content. Previous studies have attempted to quantify NMPs in organisms,^69,70^ but fecal pellet collection can lead to NMP losses. Given the importance of fecal pellets in the vertical transport of NMPs, further studies are needed to accurately quantify NMP flux in fecal pellets.

Interestingly, we observed that as diatom supply decreased, the fluorescence of NMPs in fecal pellets increased, suggesting that an increase in the proportion of NMPs in *P. crassirostris* intake under lower food availability. When food supply was abundant, fecal pellets appeared fuller with more diatom digestive residues. Cheng et al.^23^ reported higher intake rates and *in vivo* accumulation of NMPs in copepods under low diatom supply. This indicates that both NMPs and diatoms can be ingested simultaneously; however, reduced diatom availability may drive copepods to filter more water, leading to increased NMP ingestion.^71,72^

In the absence of diatoms, fecal pellets contained minimal digestive residues, with NMPs encapsulated in translucent periphyton membranes. Additionally, fecal pellets from treatment groups exposed to 1000 and 5000 μg/L NMPs with low or no diatom supply were softer and more fragile. The disruption of peritrophic membranes by NMPs, along with their repackaging, emphasizes the complex interplay between copepod digestion and defense mechanisms. NMPs may trigger false sensations and stimulate peritrophic secretion, consuming energy and potentially causing adverse effects.^73,74^ However, the peritrophic membrane also serves a protective role, facilitating NMP excretion and suggesting a defensive mechanism. Similar phenomena have been observed in krill and langoustine.^75–77^ While the peritrophic membrane helps maintain the integrity of copepod fecal pellets during settling, high NMP exposure might compromise membrane structure, leading to increased fecal pellet fragmentation.^28,29^

### Fecal Pellets Excreted by *P. crassirostris* under NMP Exposure

To quantify the effects of NMPs exposure on copepod fecal pellet size and production, we measured the dimensions of all fecal pellets that settled to the bottom after 24 h of exposure and calculated their volume, as shown in Figure 3, Figure S5–S7 and Table S3–S4. The observed lengths and widths of the fecal pellets ranged from 10.1 to 276.9 μm and 3.8 to 6.9 μm, respectively. Overall, the size of fecal pellets was significantly influenced by NMP size, concentration, and diatom supply. In the absence of microplastics, the mean length, width, and volume of copepod fecal pellets were 124.2 ± 42.5 μm, 20.1 ± 4.6 μm, and 29,628.2 ± 17,758.5 μm^3^, respectively. When copepods were exposed to NMPs, the volume of fecal pellets decreased compared to controls, with reductions of 23.6% for NPs and 65% for MPs. Pellet volume tended to decrease with increasing NMP concentrations, and more incomplete particles existed, showing reductions of 27%, 35.8%, 42.3%, and 49.5% for NMPs at concentrations of 40, 200, 1000, and 5000 μg/L, respectively. Under exposure to NMPs, the frequency of volume showed negatively skewed distribution. Smaller peaks and mean volume indicated the presence of more debris, suggesting that the fecal particles wre more fragile.

**Figure 3:**
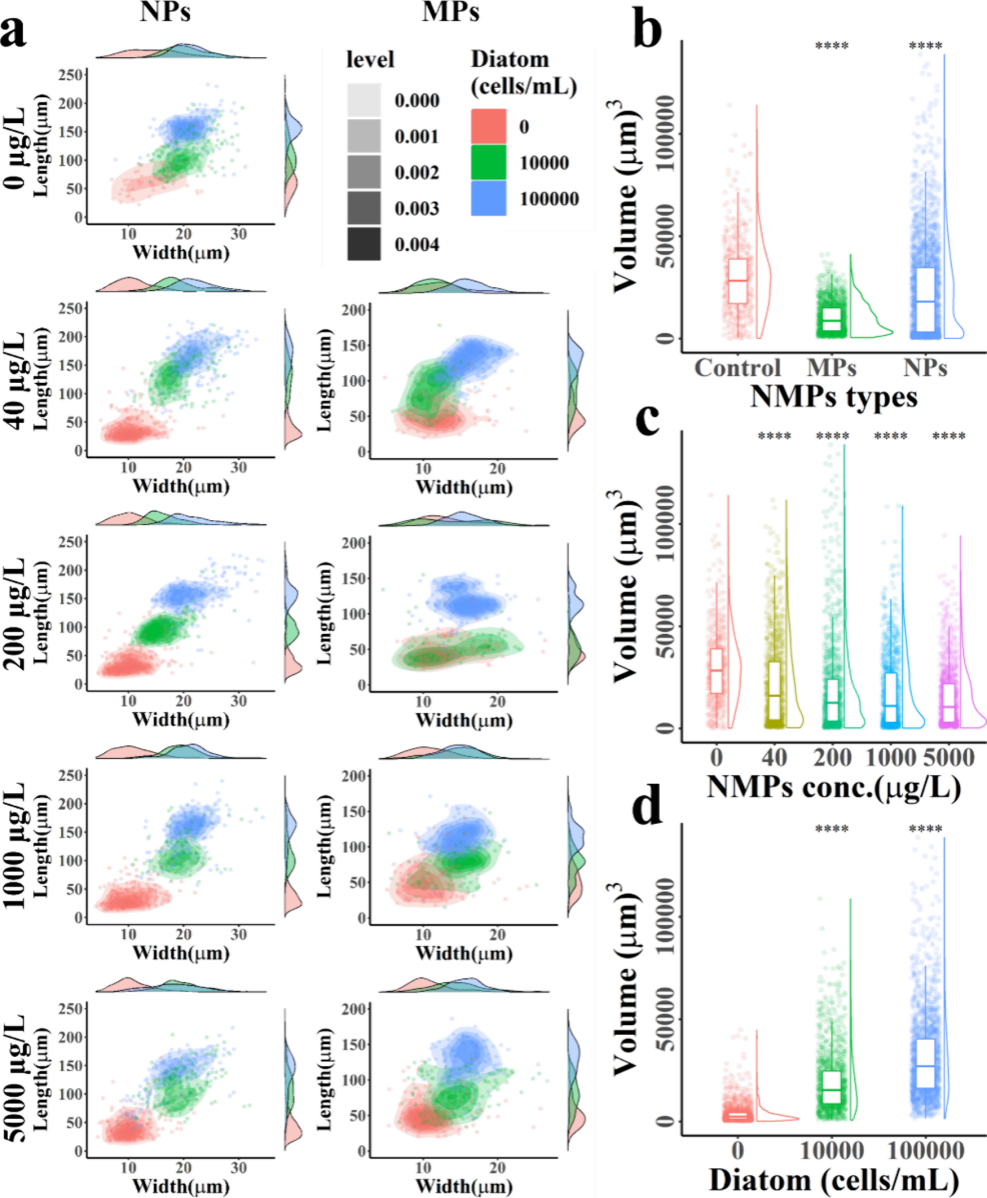
(a) Two-dimensional frequency distribution of fecal pellet size of copepods. The secondary axis is a one-dimensional frequency distribution. Data points correspond to the length and width of fecal pellets produced after exposure to a concentration gradient of the AIE-NMPs/diatom mixture. Red color shows diatom abundance of 0 cells/mL, green color shows diatom abundance of 10^4^ cells/mL, and blue color shows diatom abundance of 10^5^ cells/mL. (b) Between-group comparison of fecal pellets volume by NMPs sizes; (c) by NMPs concentrations; (d) by diatom supply. Data points are calculated volume of fecal pellets.

The availability of diatoms also resulted in significant changes in fecal pellet size, with distinct clusters observed in the two-dimensional distribution of length and width. The treatment group without diatom supply produced fecal pellets with an average length of only 45.6 ± 23.6 μm, which was smaller than those from the groups supplied with diatoms (100.6 ± 34.2 μm for 10^4^ cells/mL and 142.5 ± 34.4 μm for 10^5^ cells/mL). The width followed a similar trend, reflecting the influence of food availability on fecal pellet morphology.

Previous ecological studies have demonstrated that the quality and quantity of food are key factors influencing fecal pellet size. Besiktepe & Dam^78^ reported that the volume of fecal pellets from the copepod *A. tonsa* increased curvilinearly with diatom supply, peaking at diatom concentrations ranging from 50 to 150 μgC/L. Additionally, fecal pellet volume was found to be larger when copepods were fed ciliates and diatoms compared to flagellates.^30^ Dagg & Walser^79^ also noted an increase in fecal pellets size with higher food concentrations. However, the impact of NMP exposure on fecal pellets remains controversial. Cole et al.^29^ found no significant effect of MPs on the size of *C. helgolandicus* fecal pellets, although there was an increased tendency for fragmentation. In contrast, Rodríguez-Torres et al.^28^ concluded that exposure to environmental concentrations of MPs had no measurable effect. Conversely, Shore et al.^33^ reported that exposure to NMPs reduced the size of fecal pellets from *A. tonsa* by 2.29-fold.

Our results suggest that exposure to varying sizes and concentrations of NMPs significantly affects fecal pellet size. NMPs could stimulate the copepod’s digestive tract, leading to a reduced ingestion rate and smaller fecal pellets.^23^ Reduced fecal volume may be due to greater fragility, as NMPs mixture affected the stability of fecal particle structure.^29^ This fragility may arise from a reduction in organic matter that normally acts as an adhesive, coupled with the disruption of the peritrophic membrane by NMPs. Increased fragmentation could result in more carbon being retained in the upper layers of the ocean.^34,80^

It is important to note that our study only focused on spherical PS beads, which does not fully represent the diverse range of NMPs in nature.^57,81^ Future research should investigate the effects of different shapes of NMPs and the implications of long-term exposure on the stability of fecal particles, as well as the efficiency of vertical transport and degradation

### Fecal Pellet Production

Fecal pellet production by *P. crassirostris* exhibited varying patterns under different NMP sizes, concentrations, and diatom supply conditions, as shown in Figure 4. In the control group, where no diatoms or NMPs were supplied, only minimal amounts of fecal pellets were collected (0.13 ± 0.10 μm^3^ × 10^6^/ind./d), likely stemming from previously ingested food. When only NMPs were provided, fecal pellet production did not increase with rising NP concentrations (0.11 ± 0.07 μm^3^ × 10^6^/ind./d at 5000 μg/L NPs), indicating that copepods were not actively ingesting NPs. In contrast, production rates increased under MPs exposure (1.00 ± 0.30 μm^3^ × 10^6^/ind./d at 5000 μg/L MPs). With a supply of 10^4^ diatoms, fecal pellet production under NMP exposure initially decreased before increasing with higher concentrations. The highest fecal pellet production was observed in the absence of NMPs and with high diatom supply (4.58 ± 0.87 μm^3^ × 10^6^/ind./d). Notably, the fecal pellet production rate decreased with increasing NP concentrations, while it showed a decrease followed by an increase in response to MPs exposure.

**Figure 4:**
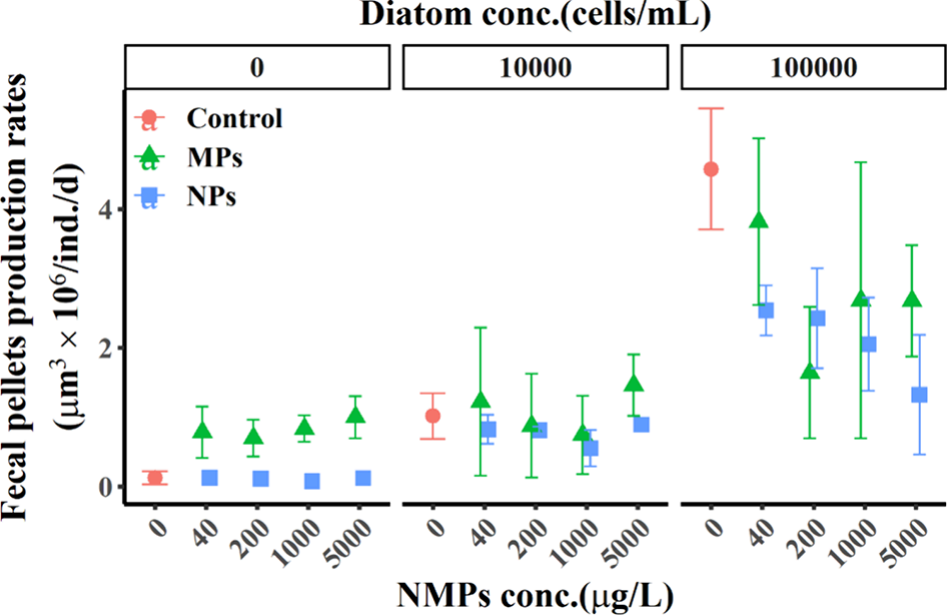
Production rate of fecal pellets. Diatom-fed controls are shown in red, MPs exposure in green, and NPs exposure in blue. Error bars represent standard deviation.

The feeding selectivity of copepods may help explain the observed patterns in fecal pellet production. Copepods utilize chemo- and mechanoreceptors to selectively ingest particles.^73^ When a greater number of NPs were introduced into the system, the overall particle count increased, but fecal pellet production did not rise. This could indicate a low capture efficiency of copepods for NPs. In contrast, exposure to MPs resulted in an increased fecal pellet production rate, reflecting effective capture of these larger particles.^23^ Rodríguez-Torres et al.^82^ also demonstrated a significant increase in fecal pellet production at high concentrations of MPs combined with low food supply, a finding supported by our previous study.^52^ Additionally, exposure of the copepod *C. helgolandicus* to NMPs was found to reduce the consumption of similarly sized and shaped algae.^27^ This suggests that NMPs may extend the processing time required for copepods to discriminate and reject NMPs, ultimately leading to a reduced ingestion rate and, consequently, lower fecal pellet production.^32^

It is important to note that our method counts all pellets settling to the bottom. Some fecal pellets may be damaged, and fragments could be dislodged during excretion and suspension, potentially leading to an underestimation of fecal pellet production in the presence of NMPs and low diatom supply.^33^ Nonetheless, the data reflect the biomass settling from the surface layer to deeper seawater. Liu et al.^83^ reported that *P. crassirostris* produced approximately 30 pellets per copepod per day with an algal supply of 1000 μg C/L, while McKinnon & Klumpp^84^ found a yield of 33 pellets per copepod per day, which aligns closely with our findings. Copepod fecal pellets are estimated to contribute approximately 40% of the vertical particulate carbon flux.^85,86^ Reduced fecal pellet production could increase the risk of carbon release back to the atmosphere, threatening nutrient availability for benthic organisms.^26^

### Modeling the Sinking of Copepod Fecal Pellets

We for the first time quantitatively revealed the setting dynamic of different sizes of fecal pellets produced by *P. crassirostris* and examined the effects of two patterns of NMP admixture with a coupled CFD-solid model (Figure 5). As shown in Figure S4, the model was validated, with the results consistent with the observations and trends of the data set. Our results indicated that fecal pellets were influenced by the combined effects of gravity and buoyancy during the sinking process. Initially, the acceleration is pronounced because the fecal pellet starts from rest. As the pellet falls, changes in its orientation alter the fluid’s trailing force, causing the velocity to fluctuate within a relatively fixed range. Any variations in sinking rates may be attributed to unbalanced forces, though this effect becomes negligible over longer time scales.

**Figure 5:**
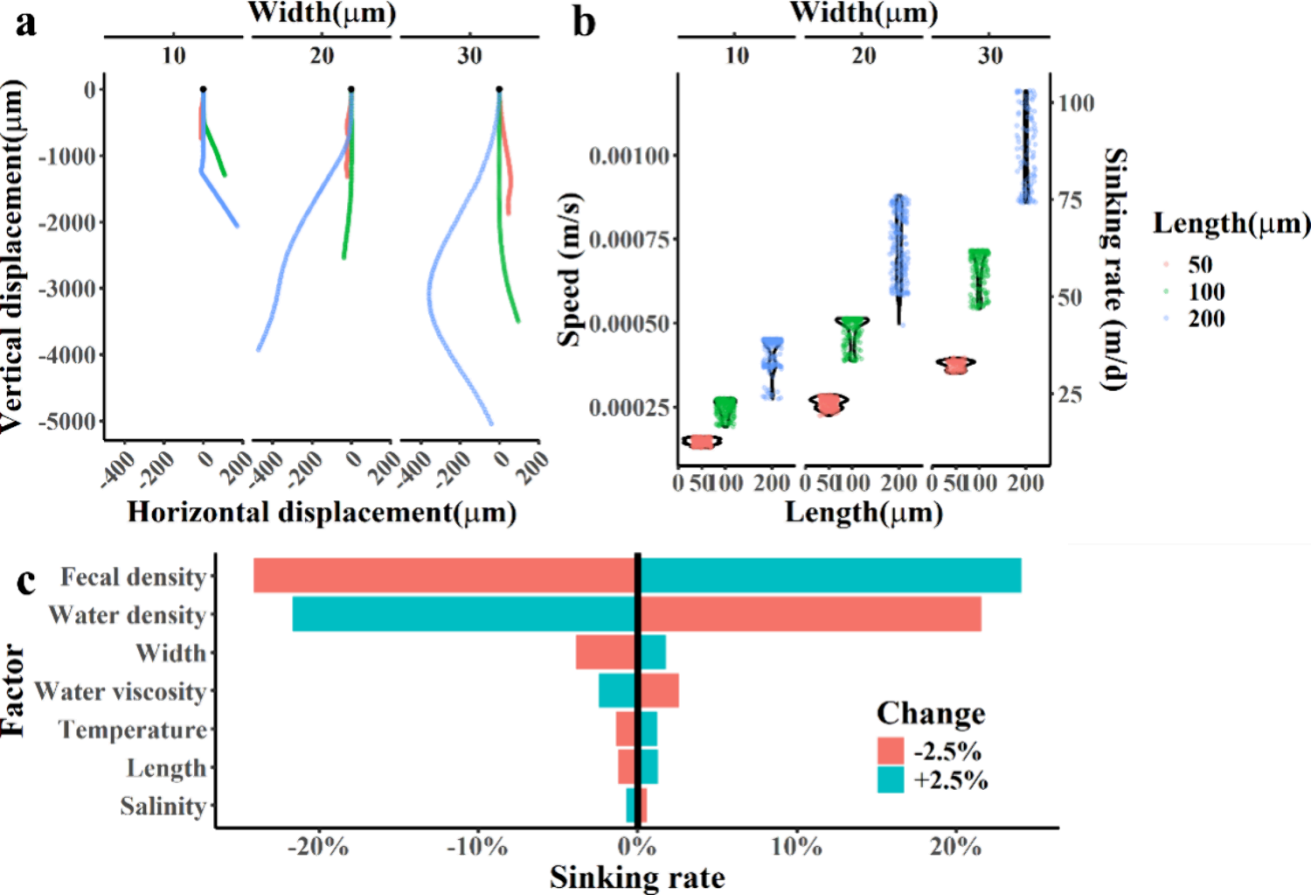
(a) Sinking trajectories of fecal pellets of different sizes. (b) Sinking rate distribution of fecal pellets. Grouped by the length and width, the colors represent the length of fecal pellets, red, green and blue for 50, 100, and 200 μm, respectively; (c) Sensitivity analysis of model, effect of ± 2.5% change of factors on sinking rate.

The modeled sinking rates ranged from 10.9 to 103.1 m/day for various fecal particle sizes, as illustrated in Figure 5. Differences were observed in the trajectories and velocities of fecal pellets falling under different density and shape conditions. As the semiaxial length and width of the fecal pellets increased, their falling velocities also increased. After the initial acceleration, the largest simulated fecal pellet (30 × 200 μm) reached a velocity of 87.4 ± 9.84 m/day. In contrast, the average falling velocities decreased by 34.3% and 62.9% for lengths of 50 and 100 μm, respectively. The slowest sinking velocity from the simulation was observed in 10 × 50 μm fecal pellets, which fell at 12.6 ± 0.84 m/day. Among the factors affecting the sinking of fecal pellets, their density is the most sensitive factor, followed by the water density. Changes in temperature and salinity lead to changes in the density and viscosity of the water, which in turn changes the sinking rate.^38^

Our modeling demonstrated that the size of fecal pellets significantly influences their sinking rate.^34^ Cole et al.^29^ found a strong positive correlation between fecal pellet descent rate and volume, both in the presence and absence of NMPs. Differences in sinking rates among fecal particles can lead to varying environmental fates. Small et al.^34^ suggested that particulate carbon in deeper water columns primarily originates from larger copepods rather than smaller ones like *P. crassirostris*. This is attributed to the fact that small copepods produce smaller fecal particles that sink more slowly.^79^ Estimates indicate that the sinking rates of fecal particles in small copepods are around 100 m/d, while larger copepods may approach 1000 m/d.^34^ Although small copepods may degrade before reaching carbon sequestration depths, they still contribute particulate carbon to the upper water column^87^. For the motion of larger scale particle populations, Lagrangian particle simulations with a random walk term are commonly used.^5^ Although diffusion is not applicable in this individual fecal pellet model, horizontal motion also indicates random walk behavior due to turbulence. The idealized hydrostatic model is inaccurate due to ocean currents, but an exploration of the influencing factors is informative.

Our results indicate that exposure to NMPs can slow the sinking rate of copepod fecal particles by reducing their volume (Figure 6). Additionally, this exposure increases the likelihood of erosion, fragmentation, and microbial degradation during the sinking process. The admixture of NMPs in copepod fecal pellets also altered their density. In cases of uniform admixture, the sinking rate increased as the percentage of polystyrene (PS) in the pellet rose. A 20 × 100 μm fecal pellet without NMPs had a sinking velocity of 39.1 ± 1.43 m/d, which decreased by 2.37 m/d when 6.25% PS was added. This change corresponds to an increase of 8.34 days in the time required to reach benthic organisms at average seabed depths of approximately 4000 m.^88^ Similar trends were observed at a 50% admixture, where the sinking velocity decreased by 34%, resulting in a delay of 59.2 days to reach the seabed. Nonuniform NMPs also influenced the sinking posture. For NMPs with densities of 950 and 1050 kg/m^3^, the descent velocity was reduced by 31.8% and 18.6%, respectively. Conversely, including the heavier 1200 kg/m^3^ NMP increased the sinking rate by 12.0%. The vertical rate distributions indicated that fecal particles tended to fall more vertically in this scenario, thereby reducing fluid resistance.

**Figure 6:**
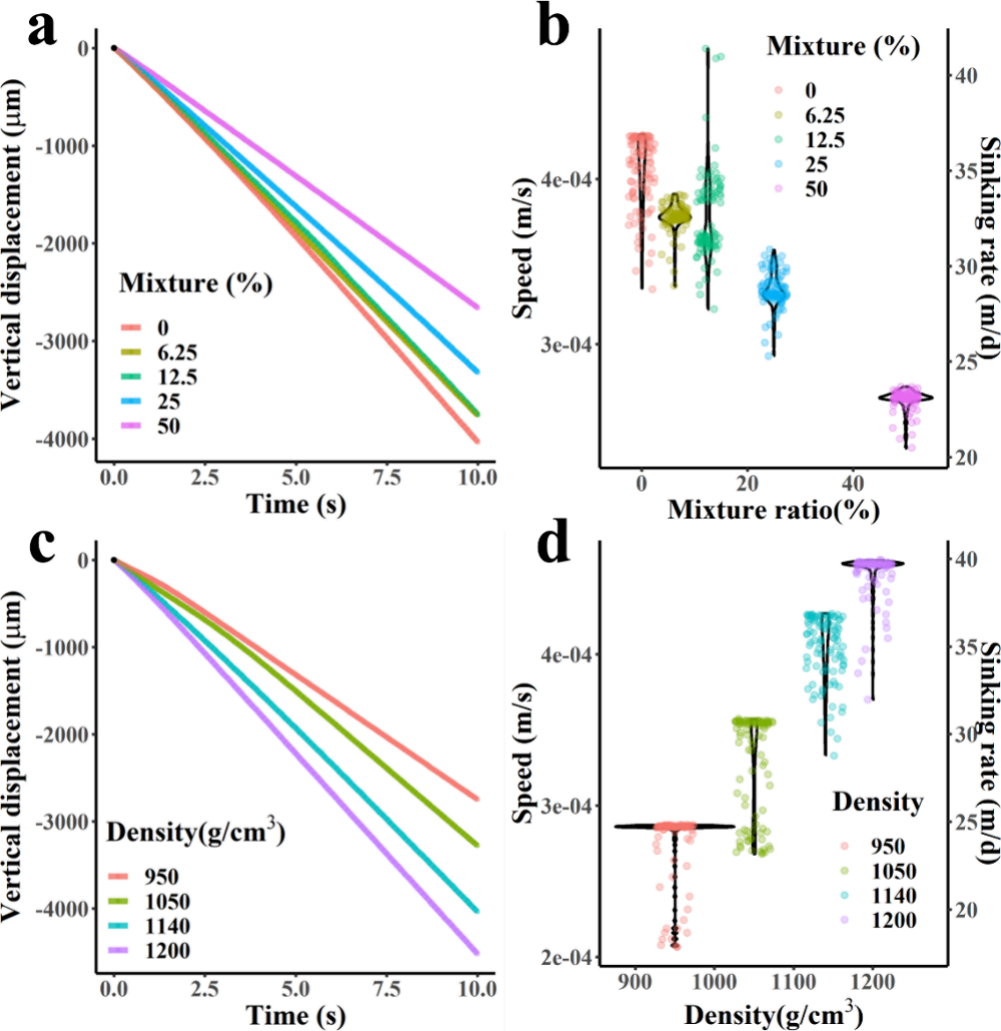
(a) Vertical displacement of fecal pellets contaminated with different ratios of PS over time. (b) Sinking rate distribution of fecal pellets under uniform admixture. (c) Vertical displacement of fecal pellets contaminated with different densities of NMPs over time, and (d) sinking rate distributions under nonuniform admixture.

### Effects of NMPs on Fecal Particle Sinking

The impact of density alterations on fecal particle sinking has been explored in previous ecological studies, considering factors like mineralized phytoplankton and lithogenic particulate matter.^31,83,89^ The influence of NMPs on fecal pellets sinking rates has also been investigated. For instance, Coppock et al.^27^ found that fecal pellets containing low-density polyethylene sink significantly slower than controls, while fecal pellets with high-density NMPs exhibited increased sinking rates. Conversely, Rodríguez-Torres et al.^28^ did not observe a significant effect of MPs on sinking rates. In natural environments, NMPs display heterogeneous spatial and temporal distributions.^57,81^ Generally, low to neutral densities of NMPs are more prevalent in surface waters, where they are more likely to be ingested by copepods, and decrease with depth. Our findings suggest that neutral and positively buoyant plastics can retard the sinking of fecal particles, whereas high-density plastics may promote sinking but are less likely to be consumed by copepods due to their rapid descent from the upper water column. Slower-sinking pellets remain in the euphotic zone longer, prolonging exposure to surface-layer microbes and increasing remineralization rates.^26^ Fragmentation of NMP polluted fecal pellets also facilitates microbial access to internal organic matter.^67^ This dual effect—delayed sinking and enhanced degradation—alters the spatial and temporal patterns of microbial degradation.

Additionally, fecal particles drift laterally during sinking, and collisions among pellets can lead to the formation of aggregates that sink more rapidly.^26^ Various environmental factors, including temperature, salinity, viscosity, turbulence, diffusion, and the degradation of the pellets, also influence the sinking rate of particulate carbon.^26^ Given that the ocean’s water column is never entirely static, hydrodynamic factors can further affect the sinking process. Accurately assessing intrinsic sinking rates is crucial for understanding other hydrodynamic interactions.^90^

The advantage of utilizing numerical simulations is the ability to customize environmental variables affecting physical interactions, allowing for hypothesis testing without the complexities of real-world confounding factors. Through detailed fluid–solid coupling modeling, we revealed the dynamic behavior of fecal pellets in the marine environment, providing valuable data to support further experimental studies and environmental protection measures.

In this study, we investigated the effects of NMP size, concentration, and diatom supply on the production and settling of fecal pellets by the copepod *P. crassirostris*. We visualized the distribution of NMPs in fecal pellets, measured the size and productivity of these pellets, and developed a numerical model to simulate their settling process in the water column. The results indicate that NMPs influence the vertical transport processes of the biological carbon pump in multiple ways. Both NPs and MPs are incorporated into fecal pellets by *P. crassirostris*, exhibiting uniform and heterogeneous distributions, respectively. NMPs reduce the size and integrity of copepod fecal pellets, leading to smaller sizes and slower sinking rates. Furthermore, NMPs alter the density of fecal pellets, which further affects sinking rates. Importantly, exposure to NMPs reduces the consumption of particulate organic carbon (POC) by copepods, thereby decreasing fecal pellet production. In the context of NMP contamination, the transport of nutrients to the seafloor becomes more challenging, limiting their availability in upper layers of the ocean. This study provides new insights into the vertical transport of copepod fecal pellets under NMP exposure, contributing to a better understanding of the risks and environmental fate of NMPs in marine ecosystems. The findings suggest that NMP pollution may have long-term adverse effects on the marine biological carbon pump.

## Supplementary Material



## Notes

Scanning electron microscope (SEM) images of the surface morphology of NMP particles; A brief diagram of the exposure protocol; Volume comparison of copepod fecal pellets before and after incubation; Validation of the model; Length, width and volume of the obtained fecal pellets for each treatment; Settings of the 27 treatments in the experiments; Model parameters and sensitivity analysis; Between-group comparisons of copepod fecal pellet length, width, and volume (PDF)
