# Supplementary material for: Modeling the Vertical Transport of Copepod Fecal Particles under Nano/Microplastic Exposure
Source: Environ Sci Technol. 2025 Mar 28;59(13):6610–22. doi: 10.1021/acs.est.5c01967 (PMC12709576; doi:10.1021/acs.est.5c01967)
Supplement: Supplementary file 1 [file es5c01967_si_001.pdf]

Supporting Information

Modeling the Vertical Transport of Copepod Fecal Particles Under Nano/Microplastic Exposure

Zipei Dong[a,b] and Wen-Xiong Wang[a,b*]

[a]*School of Energy and Environment and State Key Laboratory of Marine Pollution, City University of Hong Kong, Kowloon, Hong Kong, China*

[b]*Research Centre for the Oceans and Human Health, City University of Hong Kong Shenzhen Research Institute, Shenzhen 518057, China*

*Corresponding author, email: wx.wang@cityu.edu.hk

Number of Pages: 13

Number of Figures: 7

Number of Tables: 3

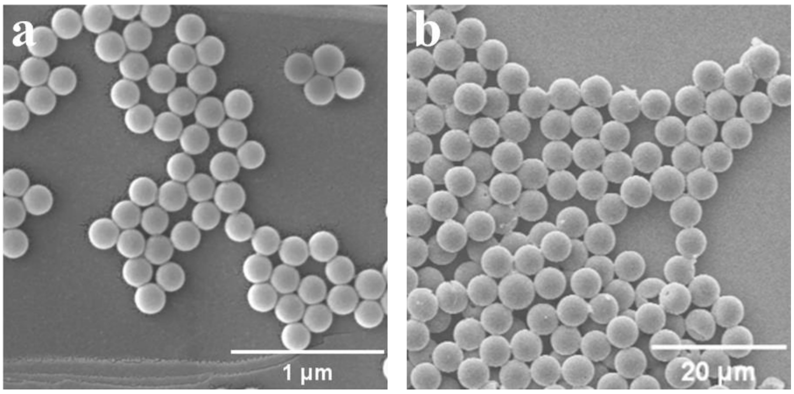

Fig. S1. Scanning electron microscope (SEM) images of the surface morphology of the NMP particles. (a) NPs; (b) MPs. The NMP suspension (100 mg/L) was dried on silicon wafers at 50 °C after 20 min of sonication, and the surface morphology was subsequently examined using SEM.

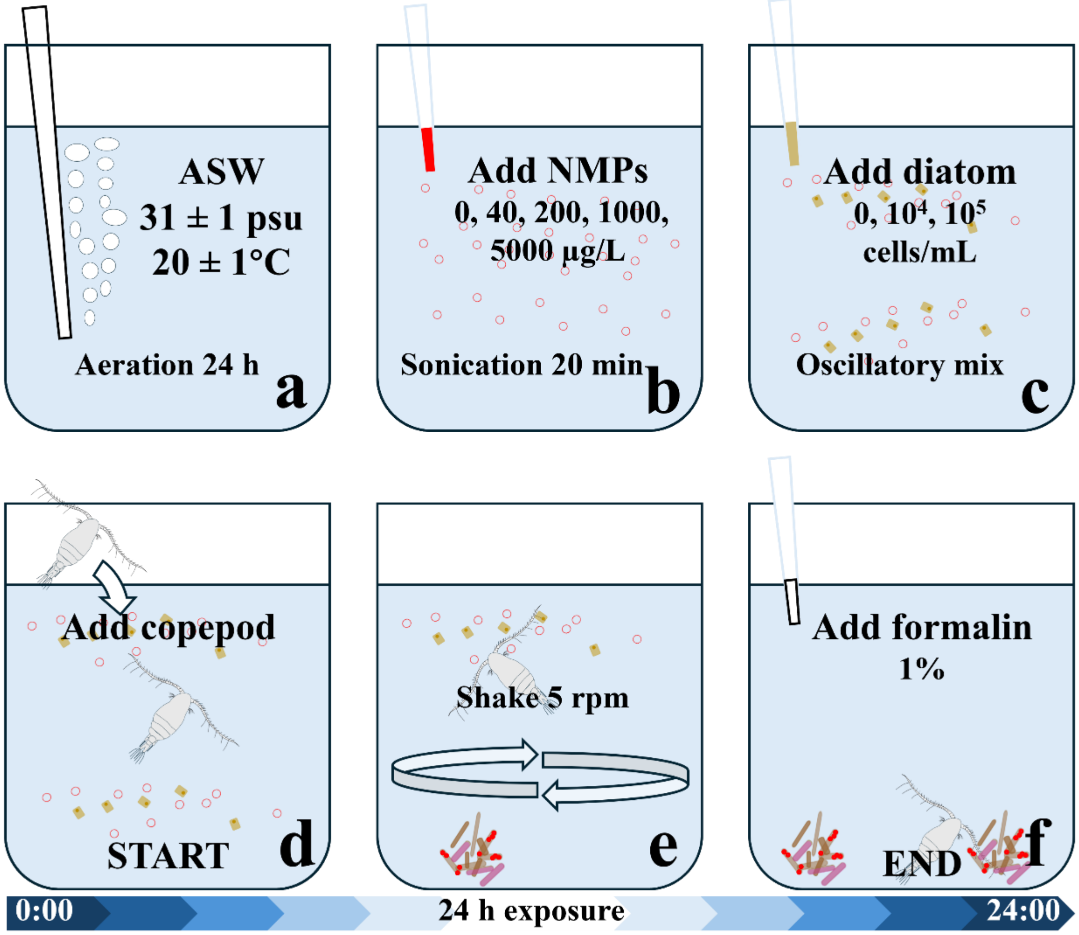

Fig. S2. A brief diagram of the exposure protocol. (a) Artificial seawater with a salinity of 31 ± 1 psu and a temperature at 20 ± 1°C was prepared and aerated for 24 h; (b) NMPs were added and then sonicated for 20 min; (c) Diatoms were then added and mixed well; (d) Add copepod and start exposure experiment at 0:00; (e) Shaking at 5 rpm during the 24-h exposure period; and (f) Add formalin (1% final concentration) at 24:00, end the exposure.

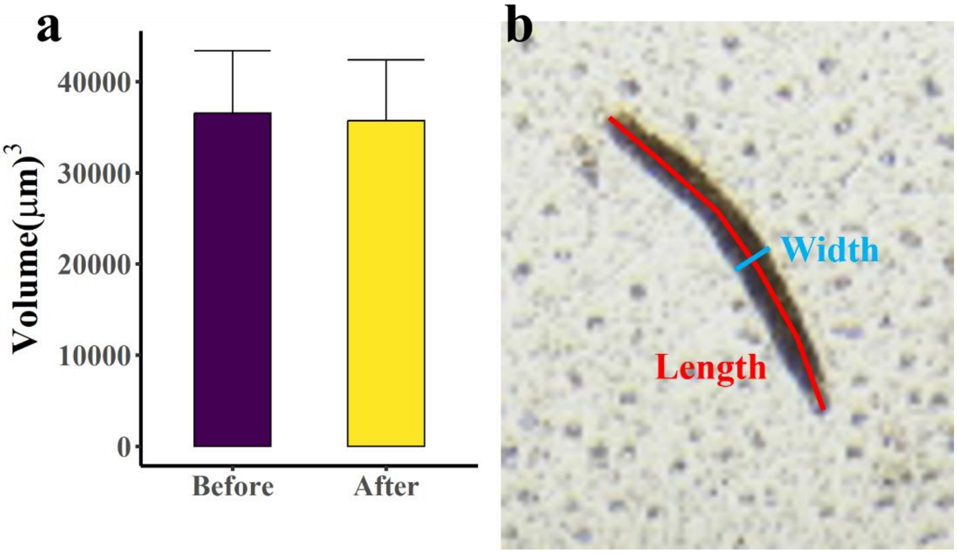

Fig. S3. (a) Volume comparison of copepod *Parvocalanus crassirostris* fecal pellets (n=10) before and after incubation for 2 hours in 1% formalin-seawater solution at 20 °C. After incubation, the volume was reduced by 2.3%, (p value = 0.63, not significant). (b) Scheme for measuring the length, width of copepod fecal pellets. For pellets exhibiting curvature, the length was determined as the longest distance along the axis using the folded line approximation.

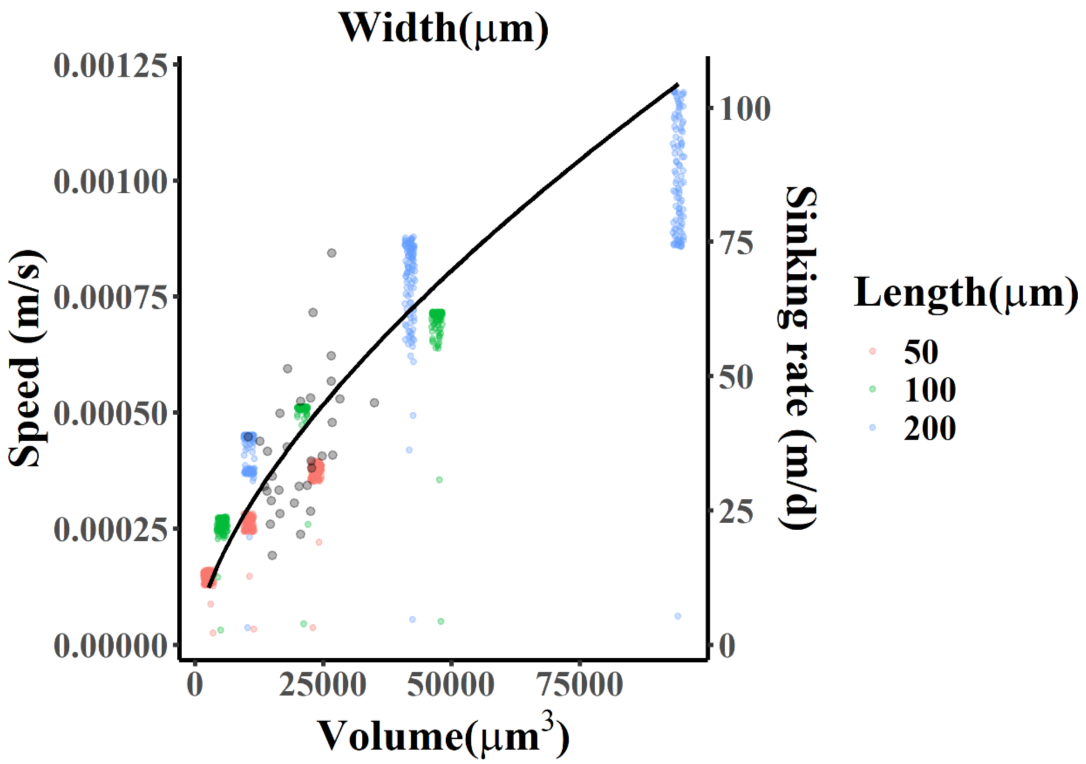

Fig. S4. Validation of the model. The simulated sedimentation rate distributions were compared with the dataset provided by Balch, W. M. & Fields, D. *(1)* (https://www.bco-dmo.org/dataset/720671). This dataset contains sizes and measured sinking rates of fecal pellets produced by the copepod *Acartia tonsa* fed on the alga *Pleurochrysis carterae* at different time points *(2)*. Black translucent dots indicate observed data. The solid black line indicates a regression performed on this data set with the results shown in Eq. S1. Sinking rates of fecal particles are grouped by length and width, with colors representing the length of the long axis and red, green, and blue representing 50, 100, and 200 µm, respectively. The horizontal coordinate is the volume of fecal particles, which is approximated as an ellipsoid computed.

$$\log S = 0.6389 \log V - 1.1594, \qquad R^2 = 0.5776 \qquad (S1)$$

Where, S is the sinking rate (m/d), and V is the Volume of fecal pellets (µm³).

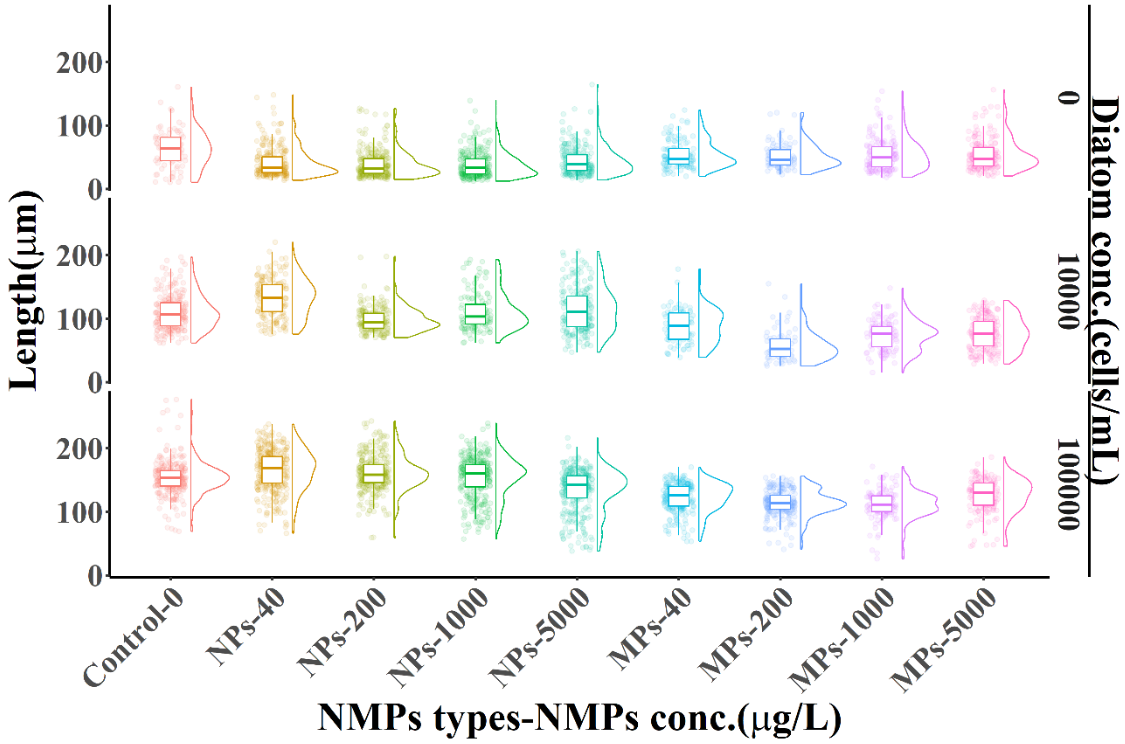

Fig. S5. The length of the obtained fecal pellets for each treatment was measured. The horizontal coordinate represents the combination of NMPs type and concentration, the vertical coordinate represents the length, and the secondary vertical coordinate is the concentration of diatom supply. Data for each treatment are presented as points, box-plot and density distribution. Points represent measured data.

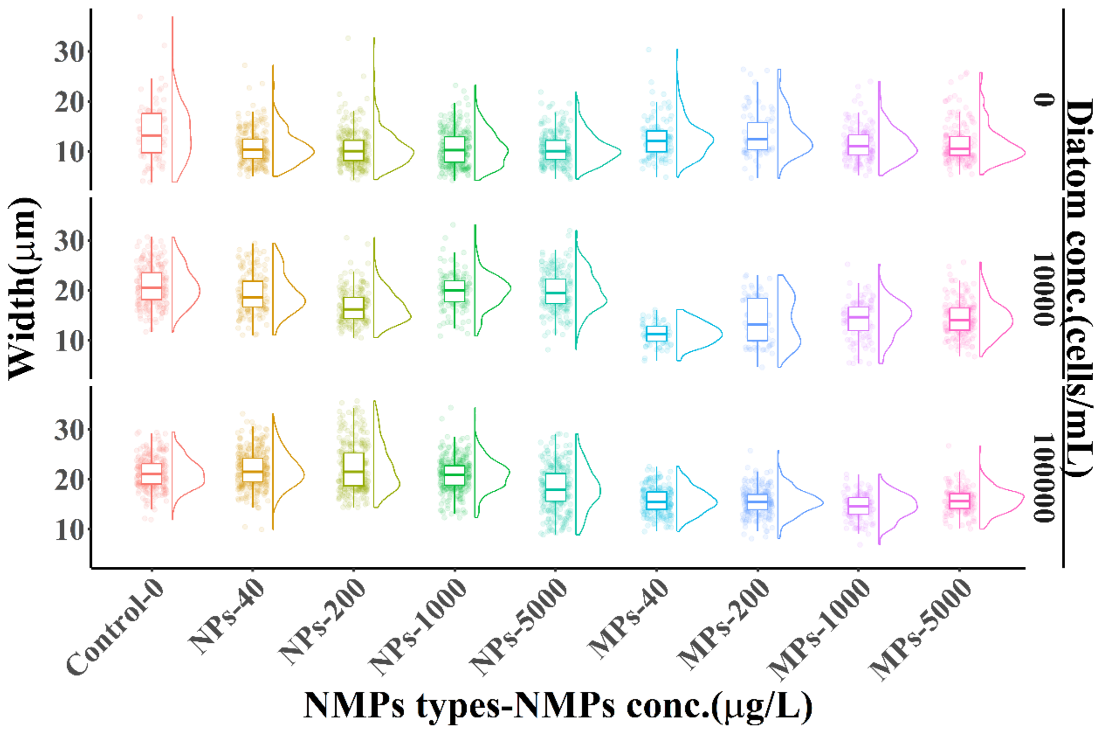

Fig. S6. The width of the obtained fecal pellets for each treatment was measured. The horizontal coordinate represents the combination of NMPs type and concentration, the vertical coordinate represents the width, and the secondary vertical coordinate is the concentration of diatom supply. Data for each treatment are presented as points, box-plot and density distribution. Points represent measured data.

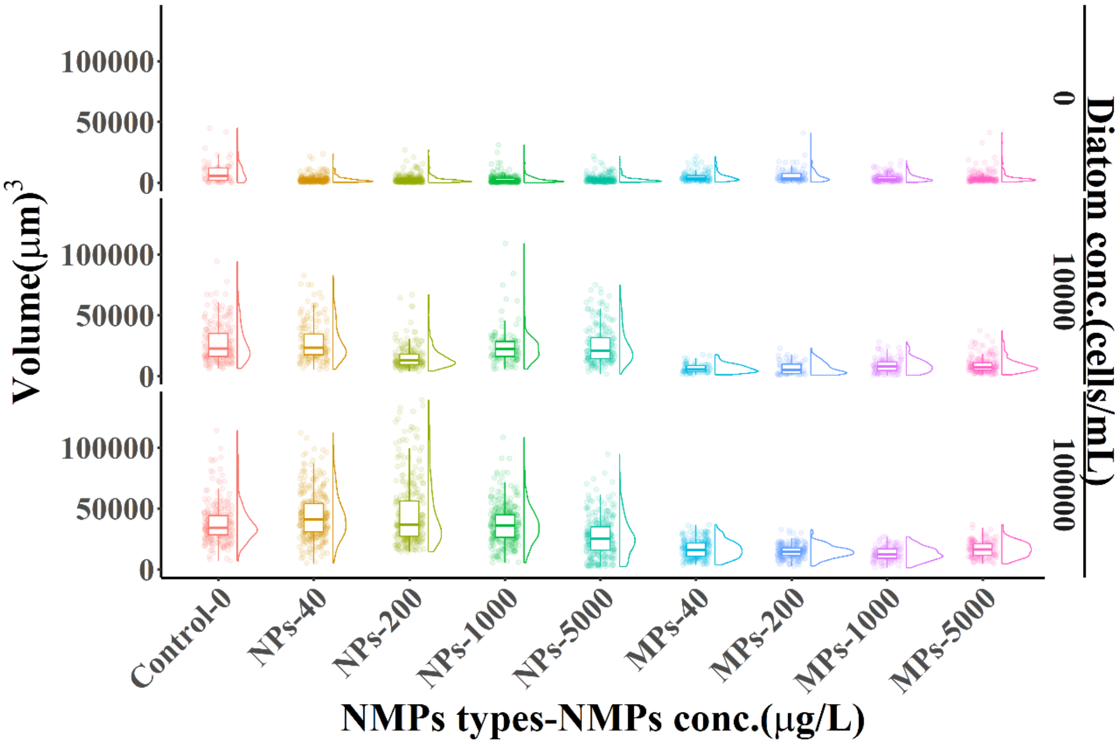

Fig. S7. The volume of the obtained fecal pellets for each treatment was measured. The horizontal coordinate represents the combination of NMPs type and concentration, the vertical coordinate represents the volume, and the secondary vertical coordinate is the concentration of diatom supply. Data for each treatment are presented as points, boxplot and density distribution. Points represent measured data.

Table S1. Settings of the 27 treatments in the experiments. Variables are nano- and microplastics (NMPs) types (Control, NPs, MPs), diatom supply (0, 10^4, 10^5 cells/mL) and NMPs concentration (0, 40, 200, 1000, 5000 µg/L).

No.	Treatments	NMPs types	Diatom supply (cells/mL)	NMPs conc. (µg/L)
1	Control-0-0	Control	0	0
2	Control-10^4-0	Control	10^4	0
3	Control-10^5-0	Control	10^5	0
4	NPs-0-40	NPs	0	40
5	NPs-0-200	NPs	0	200
6	NPs-0-1000	NPs	0	1000
7	NPs-0-5000	NPs	0	5000
8	NPs-10^4-40	NPs	10^4	40
9	NPs-10^4-200	NPs	10^4	200
10	NPs-10^4-1000	NPs	10^4	1000
11	NPs-10^4-5000	NPs	10^4	5000
12	NPs-10^5-40	NPs	10^5	40
13	NPs-10^5-200	NPs	10^5	200
14	NPs-10^5-1000	NPs	10^5	1000
15	NPs-10^5-5000	NPs	10^5	5000
16	MPs-0-40	MPs	0	40
17	MPs-0-200	MPs	0	200
18	MPs-0-1000	MPs	0	1000
19	MPs-0-50000	MPs	0	5000
20	MPs-10^4-40	MPs	10^4	40
21	MPs-10^4-200	MPs	10^4	200
22	MPs-10^4-1000	MPs	10^4	1000
23	MPs-10^4-5000	MPs	10^4	5000
24	MPs-10^5-40	MPs	10^5	40
25	MPs-10^5-200	MPs	10^5	200
26	MPs-10^5-1000	MPs	10^5	1000
27	MPs-10^5-5000	MPs	10^5	5000

Table S2. Model parameters and sensitivity analysis. Values of parameters used in the model, including fecal density, density and dynamic viscosity of water, length and width of fecal pellets, ranked in order of sensitivity magnitude from top to bottom. The sensitivity analysis used a one-way approach to compare the effects of a 2.5% increase and decrease in a single variable on sinking rates, using the parameters listed below.

	Model	+2.5%	-2.5%
Fecal density (kg/m^3)	1140.00	1168.50	1111.50
Water density (kg/m^3)	1021.51 *(3)*	1047.05	995.98
Width (μm)	10.00	10.25	9.75
Water viscosity (kg/m/s)	0.001064 *(3)*	0.001091	0.001037
Length (μm)	50.00	51.25	48.75
Salinity(ppt)	31	31.755	30.225
Temperature(°C)	20.0	20.5	19.5

Table S3. Between-group comparisons of copepod fecal pellet length, width, and volume. The groups with 0 cells/mL diatom supply were used as the reference group. p-values and significance markers with respect to the reference group are shown for each group. +, / and - represent significant increase, non-significant and significant decrease respectively. The number of + and - from 1 to 4 represent p-values <0.05, <0.01, <0.001, and <0.0001, respectively.

Comparison	Diatom, cells/mL	Control-0	MPs-40	MPs-200	MPs-1000	MPs-5000	NPs-40	NPs-200	NPs-1000	NPs-5000
Length	0	1	1	1	1	1	1	1	1	1
	10000	<2e-16<br>++++	1.00E-15<br>++++	0.075<br>/	1.80E-12<br>++++	<2e-16<br>++++	<2e-17<br>++++	<2e-18<br>++++	<2e-19<br>++++	<2e-20<br>++++
	100000	<2e-16<br>++++	<2e-16<br>++++	<2e-16<br>++++	<2e-16<br>++++	<2e-16<br>++++	<2e-16<br>++++	<2e-16<br>++++	<2e-16<br>++++	<2e-16<br>++++
Width	0	1	1	1	1	1	1	1	1	1
	10000	<2e-16<br>++++	0.08<br>/	0.86<br>/	<2e-16<br>++++	<2e-16<br>++++	<2e-16<br>++++	<2e-16<br>++++	<2e-16<br>++++	<2e-16<br>++++
	100000	<2e-16<br>++++	<2e-16<br>++++	1.90E-08<br>++++	6.30E-11<br>++++	2.70E-15<br>++++	<2e-16<br>++++	<2e-16<br>++++	<2e-16<br>++++	<2e-16<br>++++
Volumn	0	1	1	1	1	1	1	1	1	1
	10000	<2e-16<br>++++	0.00016<br>+	0.32<br>/	<2e-16<br>++++	<2e-16<br>++++	<2e-16<br>++++	<2e-16<br>++++	<2e-16<br>++++	<2e-16<br>++++
	100000	<2e-16<br>++++	<2e-16<br>++++	<2e-16<br>++++	<2e-16<br>++++	<2e-16<br>++++	<2e-16<br>++++	<2e-16<br>++++	<2e-16<br>++++	<2e-16<br>++++

Table S4. Between-group comparisons of copepod fecal pellet length, width, and volume. The groups with 0 µg/L NMPs concentration were used as the reference group. p-values and significance markers with respect to the reference group are shown for each group. +, / and - represent significant increase, non-significant and significant decrease respectively. The number of “+” and “-” from 1 to 4 represent p-values <0.05, <0.01, <0.001, and <0.0001, respectively, “/” represents non-significant, >0.05.

Comparison	Diatom, cells/mL	Control-0	NPs-40	NPs-200	NPs-1000	NPs-5000	MPs-40	MPs-200	MPs-1000	MPs-5000
Length	0	1	1.00E-08<br>----	1.70E-11<br>----	6.70E-12<br>----	1,7e-07<br>----	0.0207<br>-	0.0032<br>--	0.0143<br>-	0.0093<br>--
	10000	1	6.00E-13<br>++++	5.6e-0.6<br>----	0.82<br>/	0.26<br>/	1.00E-07<br>----	<2e-16<br>----	<2e-16<br>----	<2e-16<br>----
	100000	1	2.50E-09<br>++++	0.00088<br>+++	0.03561<br>+	1.00E-10<br>--	<2e-16<br>----	<2e-16<br>----	<2e-16<br>----	<2e-16<br>----
Width	0	1	1.20E-05<br>----	6.00E-07<br>----	2.40E-06<br>----	1.70E-06<br>----	0.1081<br>/	0.8112<br>/	0.0012<br>--	0.0012<br>--
	10000	1	0.00012<br>---	<2e-16<br>----	0.03475<br>-	0.00437<br>--	<2e-16<br>----	<2e-16<br>----	<2e-16<br>----	<2e-16<br>----
	100000	1	0.018<br>-	0.081<br>/	0.26<br>/	<2e-16<br>----	<2e-16<br>----	<2e-16<br>----	<2e-16<br>----	<2e-16<br>----
Volumn	0	1	1.00E-09<br>----	2.10E-11<br>----	2.10E-11<br>----	1.70E-09<br>----	0.01206<br>-	0.09544<br>/	0.00016<br>---	0.00012<br>---
	10000	1	0.53<br>/	<2e-16<br>----	0.12<br>/	0.14<br>/	<2e-16<br>----	<2e-16<br>----	<2e-16<br>----	<2e-16<br>----
	100000	1	9.60E-06<br>++++	0.041<br>+	0.789<br>/	<2e-16<br>----	<2e-16<br>----	<2e-16<br>----	<2e-16<br>----	<2e-16<br>----

Reference

(1) Balch, W. M.; Fields, D. Fecal pellet sinking rate experiment and calculated density for the study: Gut dissolution of coccoliths by *Acartia*/effect on fecal pellet sinking. *Biological and Chemical Oceanography Data Management Office (BCO-DMO)*. **2017**, *12*, Version 1.

(2) White, M. M.; Waller, J. D.; Lubelczyk, L. C.; Drapeau, D. T.; Bowler, B. C., Balch, W. M.; & Fields, D. M. Coccolith dissolution within copepod guts affects fecal pellet density and sinking rate. *Sci. Rep.* **2018**, *8(1)*, 9758.

(3) El-Dessouky, H. T.; H. M. Ettouney. "Appendix A: Themodynamic Properties." Fundamentals of Salt Water Desalination. *Elsevier Science: Amsterdam, The Netherland*. **2002**.
